# Encapsulin protein MAV2054 enhances *Mycobacterium avium* virulence by promoting Cdc42-dependent epithelial cell invasion

**DOI:** 10.71150/jm.2506008

**Published:** 2025-11-30

**Authors:** Dong Ho Kim, I Jeong Jo, Min Ju Kang, Yi Seol Kim, Duyen Do Tran Huong, Kyungho Woo, Ho-Sung Park, Hwa-Jung Kim, Chul Hee Choi

**Affiliations:** 1 Department of Microbiology, School of Medicine, Chungnam National University, Daejeon 35015, Republic of Korea; 2 Translational Immunology Institute, School of Medicine, Chungnam National University, Daejeon 35015, Republic of Korea; 3 Department of Medical Science, School of Medicine, Chungnam National University, Daejeon 35015, Republic of Korea; 4 System Network Inflammation Control Research Center, School of Medicine, Chungnam National University, Daejeon 35015, Republic of Korea

**Keywords:** *Mycobacterium avium*, encapsulin nanocompartment, MAV2054, actin cytoskeleton remodeling

## Abstract

*Mycobacterium avium* complex (MAC) organisms are widespread environmental pathogens associated with chronic pulmonary infections. Although *M. avium* is known to invade epithelial cells, the molecular mechanisms underlying this process remain incompletely understood. In this study, we identified a novel role for MAVRS09815 (formerly MAV2054), a family 2A encapsulin nanocompartment shell protein, in mediating bacterial adhesion, epithelial cell invasion, and in vivo virulence. We engineered a recombinant *M. smegmatis* strain expressing MAV2054 (Ms_2054) and an *M. avium* MAV2054 deletion mutant (Δ2054). Ms_2054 exhibited enhanced epithelial invasion, whereas Δ2054 showed reduced intracellular survival. Recombinant MAV2054 protein was bound directly to human epithelial cells in a dose-dependent manner. Pretreatment of host cells with cytochalasin D or vinblastine significantly inhibited bacterial internalization, indicating that MAV2054-mediated invasion is cytoskeleton-dependent. Confocal and scanning electron microscopy revealed MAV2054-dependent membrane rearrangements during infection. Pull-down assays demonstrated that MAV2054 activates Cdc42, a key regulator of actin polymerization, with reduced activation observed in Δ2054-infected cells. In a murine intratracheal infection model, the Δ2054 exhibited significantly reduced bacterial burdens and lung inflammation compared to the wild type. These findings demonstrate that MAV2054 enhances *M. avium* virulence by promoting epithelial cell invasion through Cdc42-dependent cytoskeletal remodeling. This study reveals a previously unrecognized role for an encapsulin-like protein in host-pathogen interactions and highlights its potential as a therapeutic target in MAC infections.

## Introduction

Nontuberculous mycobacteria (NTM) comprise a diverse group of over 200 species within the genus *Mycobacterium*, excluding *Mycobacterium tuberculosis* and *Mycobacterium leprae* (Cowman et al., 2019; Falkinham, 2022). These organisms are ubiquitous in the environment and can be found in water, soil, animals, and food sources (Tan and Kasperbauer, 2021; To et al., 2020). Although generally regarded as opportunistic pathogens, NTMs are increasingly recognized as causative agents of pulmonary disease (NTM-PD), even in immunocompetent individuals (Cowman et al., 2019; Schildkraut et al., 2021; To et al., 2020). The global incidence of NTM-PD continues to rise, presenting significant diagnostic and therapeutic challenges (Lee et al., 2024; Park et al., 2022). Clinical presentation varies widely, ranging from stable cases requiring no intervention to progressive, treatment-refractory disease associated with considerable morbidity and mortality (Cowman et al., 2019). Further complicating management is the emergence of antibiotic-resistant NTM strains, which significantly hampers therapeutic success (van Ingen et al., 2012; Wu et al., 2018).

Among NTM species, the *Mycobacterium avium* complex (MAC)—including *M. avium*, *M. intracellulare*, and *M. chimaera*—is the most common cause of NTM-PD in humans (Kumar et al., 2024; Kwon et al., 2019). MAC species are facultative intracellular pathogens capable of invading and replicating within macrophages, fibroblasts, endothelial cells, and mucosal epithelial cells (Abukhalid et al., 2021; Bermudez et al., 2000). Successful infection depends on bacterial adhesion and invasion of host cell surfaces (Bermudez et al., 1995). Various bacterial surface components such as glycopeptidolipids and fibronectin attachment protein homologs mediate these interactions (Schorey and Sweet, 2008; Secott et al., 2004). However, the precise molecular mechanisms governing *M. avium* adhesion and invasion remain poorly understood.

A previous study identified the MAV2054 protein (MAVRS09815) as an inducer of apoptosis via mitochondrial dysfunction (Lee et al., 2016). Although initially annotated as major membrane protein 1, MAV2054 has since been reclassified as a family 2A encapsuling nanocompartment shell protein. Unlike eukaryotes, which possess membrane-bound organelles, prokaryotes achieve compartmentalization through protein-based structures such as bacterial microcompartments and encapsulin nanocompartments. These encapsulins are categorized into four families based on sequence homology, Pfam (protein family) membership, and genome-neighborhood composition. Notably, family 1 (ferritin-like proteins; Flp) and family 2 (desulfurase systems) encapsulins have been implicated in promoting pathogen survival, host invasion, and biofilm formation under stress conditions (Giessen, 2022). Despite these emerging insights, the specific role of encapsulin systems in mycobacterial pathogenesis remains largely unexplored.

In this study, we utilized both a MAV_2054-expressing *M. smegmatis* strain and a MAV_2054-deletion mutant of *M. avium* to investigate the functional role of MAV2054 in infection. Although *M. smegmatis* harbors a highly conserved homolog of MAV_2054 (~90% amino acid identity), its functional role remains uncharacterized. Therefore, ectopic overexpression of the *M. avium* MAV_2054 gene in *M. smegmatis* may enhance or reveal virulence-related functions that are otherwise latent or minimally expressed in the native context. This approach allowed us to dissect the specific contribution of MAV_2054 to host interaction, independently of other *M. avium*-specific virulence determinants. Our comparative analyses revealed that MAV2054 enhances bacterial adhesion to and invasion of human lung epithelial cells. Notably, invasion was significantly reduced following treatment with actin polymerization inhibitors, indicating a cytoskeleton-dependent mechanism. These findings highlight MAV2054 as a key factor facilitating *M. avium* epithelial invasion and underscore its potential role in the early stages of infection.

## Materials and Methods

### Bacteria strains, growth, and culture conditions

The bacterial strains used in this study included *M. smegmatis* vector control (Ms_Vc, pVV16), MAV2054-expressing *M. smegmatis* (Ms_2054, pVV16_MAV2054), *M. avium* wild-type (WT), and the MAV2054 deletion mutant (Δ2054). All strains were kindly provided by Dr. Hwa-Jung Kim (Department of Microbiology, School of Medicine, Chungnam National University). Bacteria were cultured in Middlebrook 7H9 medium (Difco) supplemented with 10% (v/v) OADC (NAVI BIOTECH, Korea) at 37°C with shaking at 140 rpm. Bacterial growth was monitored by measuring optical density at 600 nm (OD_600_). Data from the OD_600_ value were analyzed using an unpaired *t*-test in GraphPad Prism 5 (version 5.01). Cultures were harvested, suspended in Dulbecco’s Phosphate Buffered Saline (DPBS; WELGENE, Korea), and then stored at –80°C until further use.

### Confirmation of MAV2054 expression

Recombinant *M. smegmatis* strains (Ms_Vc and Ms_2054) were cultured in 7H9 broth supplemented with 10% (v/v) oleic acid-albumin-dextrose-catalase (OADC) and kanamycin (50 μg/ml) until the OD_600_ reached 1.0. *M. avium* strains (WT and Δ2054) were similarly cultured in 7H9 broth supplemented with 10% (v/v) OADC to an OD_600_ of 1.0. Bacterial cells were collected by centrifugation at 3,000 rpm for 30 min at 4°C and subsequently lysed on ice using a probe-type sonicator (Sonics & Materials, USA) operated at 12% amplitude (AMP) for 30 min in the presence of a protease inhibitor cocktail (Thermo Scientific, USA). The lysates were fractionated into supernatant and pellet fractions by centrifugation. All protein fractions were analyzed using SDS-PAGE followed by Coomassie Brilliant Blue staining (Bio-Rad Laboratories, USA). For immunoblotting, protein samples were separated using SDS-PAGE and transferred onto a PVDF membrane. The membranes were blocked with EZBlock Chemi (ATTO, Japan), and then incubated overnight at 4°C with either an anti-His-tag or polyclonal anti-MAV2054 antibody (mouse; 1:1,000 dilution). On the following day, the membranes were incubated with appropriate Horseradish Peroxidase (HRP)-conjugated secondary antibodies for 1 h at 25°C. Detection was performed using WesternBright^®^ ECL (Advansta, USA), and chemiluminescent signals were visualized using the ChemiDoc XRS+ imaging system (Bio-Rad Laboratories, USA).

### Survival of recombinant *M. smegmatis* strains under stress conditions

Recombinant *M. smegmatis* strains (Ms_Vc and Ms_2054) were cultured in 7H9 broth supplemented with 10% (v/v) OADC until the OD_600_ reached 0.5. The cultures were then subjected to stress conditions by exposure to either acidic pH (pH 3.0) or 0.05% sodium dodecyl sulfate (SDS; Bio-Rad Laboratories). At the indicated time points (0, 2, 4, 6, and 24 h), samples were collected, serially diluted ten-fold, and then spotted onto 7H10 agar plates. Bacterial survival was calculated as the percentage of colony-forming units (CFUs) relative to untreated control cultures.

### Cell culture

Human epithelial cell lines, including A549 (lung; ATCC CCL-185), HEp-2 (laryngeal; ATCC CCL-23), and HeLa (cervical; ATCC CCL-2), were used in this study. Cells were maintained in Dulbecco’s Modified Eagle’s Medium (DMEM; WELGENE) supplemented with 10% fetal bovine serum (FBS; WELGENE) at 37°C in a humidified atmosphere containing 5% CO₂.

### Epithelial cell binding of rMAV2054

A total of 1 × 10^5^ cells (A549, HeLa, or HEp-2) were incubated at 4°C for 30 min in DMEM containing either 1 μg/ml or 5 μg/ml of recombinant MAV2054 protein (rMAV2054). After incubation, the cells were washed with DPBS and incubated with a polyclonal anti-MAV2054 antibody (mouse, 1:1,000 dilution). Following primary antibody binding, cells were stained with Alexa Fluor 488-conjugated anti-mouse IgG (Life Technologies, USA). Flow cytometric analysis was performed using a NovoCyte flow cytometer (BD Biosciences, USA), as previously described (Nho et al., 2015).

### Invasion assay

A549 cells were seeded into 24-well plates and infected with each bacterial strain (Ms_Vc, Ms_2054, *M. avium* WT, and Δ2054) at a multiplicity of infection (MOI) of 10 or 50 for 6 h at 37°C. To eliminate extracellular bacteria, cells were incubated with fresh medium containing amikacin (200 μg/ml; Sigma-Aldrich, USA) for 1 h. After treatment, the wells were washed three times with DPBS, and the cells were lysed with 0.25% Triton X-100 (Sigma-Aldrich) for 30 min at 25°C. The resulting lysates were collected, serially diluted, and plated on Middlebrook 7H10 agar. CFUs were enumerated after 3 days (*M. smegmatis*) or 7 days (*M. avium*) of incubation at 37°C.

### Scanning-electron microscopy

A549 cells were seeded into 24-well plates containing 13-mm-diameter coverslips (Thermo Scientific) and incubated with or without cytochalasin D (2 μM) or vinblastine (100 μM) for 30 min prior to infection. Cells were then infected with bacteria (Ms_Vc, Ms_2054, *M. avium* WT, or Δ2054) at an MOI of 50 for 6 h at 37°C. After infection, cells were treated with a medium containing amikacin (200 μg/ml) for 1 h to eliminate extracellular bacteria. The cells were then washed with DPBS and fixed with 4% paraformaldehyde for 24 h at 4°C. Fixed samples were washed three times with DPBS and dehydrated through a graded acetone series (30% to 100%) at 4°C. Dehydrated samples were coated with a conductive layer using an ion sputter coater (GSEM, Korea), and scanning electron microscopy analysis was performed using a Mini-scanning electron microscopy system (COXEM, Korea).

### Confocal microscopy

Each bacterial strain (Ms_Vc, Ms_2054, *M. avium* WT, and Δ2054) was stained with carboxyfluorescein diacetate succinimidyl ester (CFSE; Invitrogen, USA), as previously described (Kim et al., 2013). A549 cells were seeded onto glass coverslips and infected with CFSE-labeled bacteria for 6 h at MOI of 10. After infection, cells were washed three times with DPBS and fixed with 4% paraformaldehyde (Electron Microscopy Sciences) for 1 h at 25°C. Actin filaments were stained with Alexa Fluor 594-conjugated phalloidin (Invitrogen) for 30 min. The coverslips were mounted using VECTASHIELD Antifade Mounting Medium containing DAPI (Vector Laboratories, USA), and fluorescence images were acquired using an LSM 900 confocal microscope (Zeiss, Germany).

### Murine pulmonary infection model

Pathogen-free, six-week-old female C57BL/6 mice were purchased from Narabiotec (Korea) and randomly divided into two groups (total 6 mice, 3 mice per group). All animal experiments were approved by the Institutional Research and Ethics Committee of Chungnam National University (202410A-CNU-221) and conducted in accordance with the guidelines of the Korean Food and Drug Administration. Each group of C57BL/6 mice (n = 3) was anesthetized with 2,2,2-tribromoethanol (125 mg/kg, Sigma-Aldrich) and intratracheally infected with *M. avium* strains (either *M. avium* WT, or Δ2054 with each strain administered at 1 × 10^7^ CFU/mouse). The mice were monitored until the effects of anesthesia wore off and, upon awakening, were returned to their cages. Mice were sacrificed on the two weeks post-infection to measure the bacterial burden in the lungs. Lung samples were collected and homogenized, after which the bacteria were serially diluted with DPBS and spread onto Middlebrook 7H10 plates for enumeration. Data from the infection experiments were analyzed using an unpaired *t*-test in GraphPad Prism 5 (version 5.01) to determine the effect of MAV2054 on *M. avium* survival.

### Histopathological analysis

Lungs were excised from infected mice and fixed in 10% formalin (Sigma-Aldrich), and then embedded in paraffin. Each section was stained with acid-fast bacilli (AFB) or hematoxylin and eosin (H&E). Histopathology was performed based on previous studies (Asay et al., 2020). Area of inflammatory lesions in H&E-stained lungs was measured using by ImageJ (version 1.54i).

### Cdc42 activation assay

Cdc42 activation in infected cells was assessed using a Cdc42 Activation Assay Kit (Cytoskeleton, USA) following the manufacturer’s instructions. Briefly, A549 cells were infected with bacteria (Ms_Vc, Ms_2054, *M. avium* WT, or Δ2054) at an MOI of 10 for 1 h. Both infected and uninfected cells were washed three times with PBS and detached using a cell scraper. Cell lysates were incubated with PAK-RBD beads at 4°C on a rocking platform for 1 h. The beads were then collected by centrifugation and washed three times with assay buffer. Proteins were separated using SDS-PAGE and transferred onto PVDF membranes. Membranes were blocked with EZBlock Chemi (ATTO), incubated overnight at 4°C with an anti-Cdc42 antibody, and subsequently incubated with an HRP-conjugated secondary antibody for 1 h at 25°C. Protein bands were visualized using WesternBright^®^ ECL (Advansta). The ChemiDoc XRS+ system (Bio-Rad Laboratories) was used to detect the bands, and Image Lab software was used to quantify the bands. The GTP-Cdc42 (active form) levels were normalized to those of total-Cdc42 and the values were further normalized to the uninfected control, which was set to 1.0.

### Inhibition of bacterial uptake assay

To assess the role of the cytoskeleton in bacterial uptake, A549 cells were seeded into 24-well plates and pretreated with either cytochalasin D (2 μM; Tocris Bioscience, UK) or vinblastine (100 μM; Tocris Bioscience) for 30 min prior to infection. Cells were then infected with either *M. avium* WT or Ms_2054 at an MOI of 10 and incubated for 6 h at 37°C. Following infection, extracellular bacteria were removed by incubating the cells with fresh medium containing amikacin (200 μg/ml) for 1 h. Cells were subsequently washed three times with DPBS and lysed with 0.25% Triton X-100 for 30 min at 25°C. Cell lysates were collected, serially diluted, and then plated on Middlebrook 7H10 agar. CFUs were enumerated after 3 days (*M. smegmatis*) or 7 days (*M. avium*) of incubation at 37°C.

### Statistical analysis

All experiments were performed in triplicate or more. Statistical analyses were conducted using unpaired *t*-tests or one-way analysis of variance (ANOVA) followed by Tukey’s post hoc test using GraphPad Prism version 5.01 (GraphPad Software). Data are presented as mean ± standard error of the mean. A *p*-value < 0.05 was considered statistically significant.

## Results

### MAV2054 modulates surface morphology in Mycobacteria

**Fig. 1.**
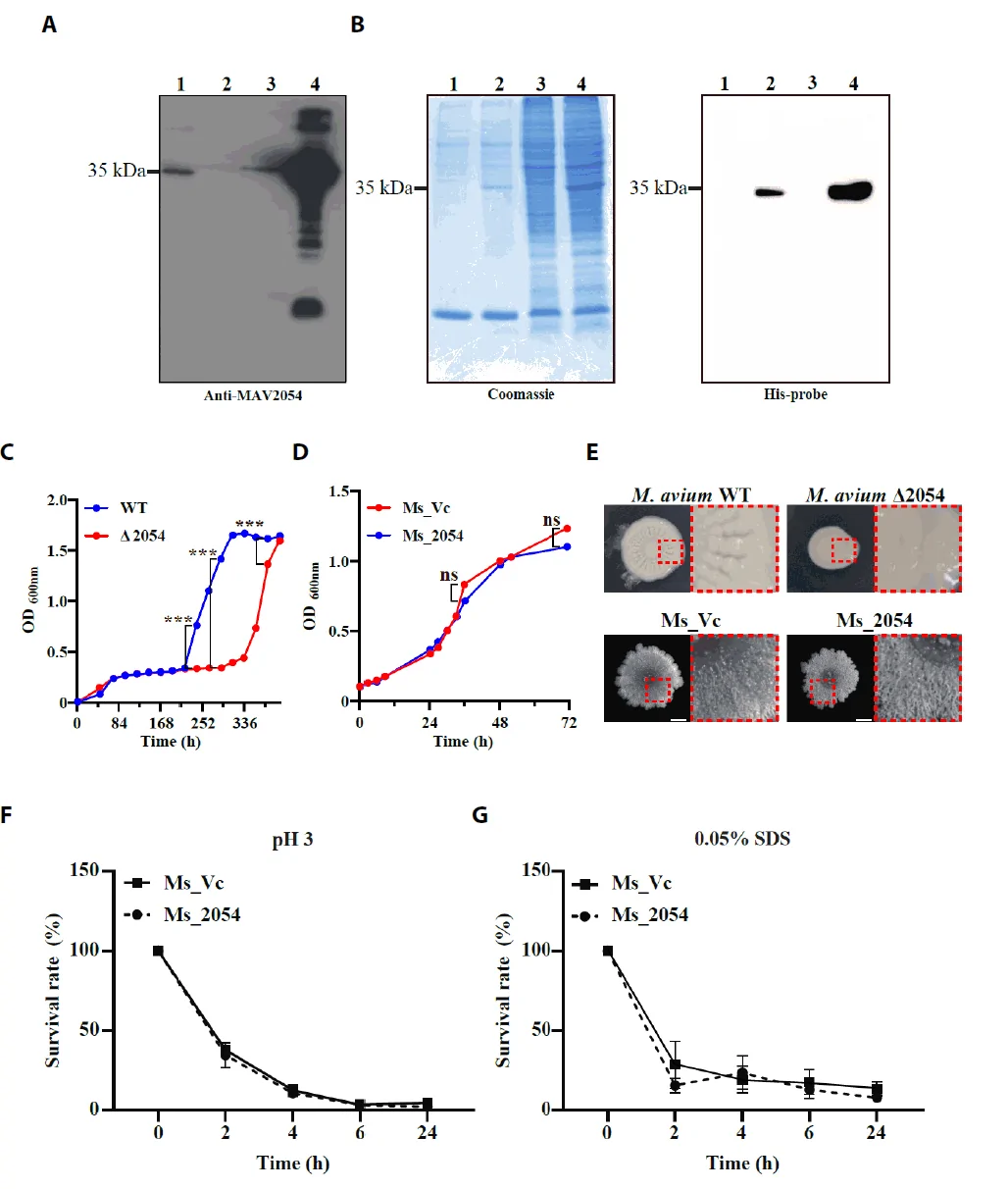
Characterization of MAV2054-expressing *M. smegmatis* strains. (A) MAV2054 expression in *M. avium* WT and the Δ2054 strains. Total protein lysates were analyzed by SDS-PAGE followed by immunoblotting using an anti-MAV2054 antibody. Lane 1: lysate of *M. avium* WT, lane 2: lysate of *M. avium* Δ2054, lane 3: cultured media of *M. avium* WT, lane 4: recombinant MAV_2054 protein. (B) Confirmation of MAV2054 expression in *M. smegmatis* strains harboring either the empty vector (Ms_Vc) or pVV16-MAV2054 (Ms_2054). Total protein lysates were analyzed by SDS-PAGE, followed by Coomassie Brilliant Blue staining and immunoblotting using an anti-His antibody. Lane 1: soluble proteins of Ms_Vc, lane 2: soluble proteins of Ms_2054, lane 3: insoluble proteins of Ms_Vc, lane 4: insoluble proteins of Ms_2054. (C) Growth curves of *M. avium* WT and Δ2054 strains cultured in 7H9 medium at 37°C. (D) Growth curves of Ms_Vc and Ms_2054 strains cultured in 7H9 medium at 37°C. Data are represented as the mean from three sets of independent experiments. (E) Colony morphology of *M. avium* and *M. smegmatis* strains grown on 7H10 agar plates. (F, G) Stress tolerance of *M. smegmatis* strains under acidic conditions (pH 3.0) (F) and 0.05% SDS treatment (G). Data are presented as mean ± standard error of mean (SEM). Statistical significance: ^***^p < 0.001. WT, *M. avium* wild-type; Δ2054, *M. avium* MAV2054 deletion mutant; Ms_Vc, *M. smegmatis* vector control; Ms_2054, MAV_2054-expressing *M. smegmatis*.

To investigate the role of MAV2054 in MAC, we generated a gene deletion mutant (Δ2054). Successful deletion was confirmed by immunoblotting (Fig. 1A). We also constructed a MAV2054 expressing *M. smegmatis* strain (Ms_2054) using the pVV16 vector. As a control, an empty vector strain (Ms_Vc) was generated by introducing the empty pVV16 plasmid into *M. smegmatis*. Expression of MAV2054 in Ms_2054 was verified by immunoblotting using an anti-His antibody recognizing the His-tag encoded by the pVV16 vector. A distinct band of approximately 35 kDa, corresponding to the expected size of MAV2054, was detected in Ms_2054 but not in Ms_Vc (Fig. 1B). For reference, recombinant MAV2054 protein was included as a size control (Fig. 1A).

To assess whether MAV2054 affects bacterial growth, we monitored the proliferation of the *M. avium* WT, Δ2054, Ms_2054, and Ms_Vc strains at 37°C. Ms_2054 and Ms_Vc showed no significant differences in growth (Fig. 1D). However, *M. avium* Δ2054 exhibited a delayed onset of exponential growth compared to the WT strain. Specifically, the *M. avium* WT entered the exponential phase approximately 220 h post-inoculation, while the Δ2054 did so around 312 h, indicating a ~90 h delay. Statistical analysis revealed that the Δ2054 strain showed significantly lower growth than the WT between 240 h and 384 h post-inoculation. Nevertheless, both strains required approximately 100 h to progress from exponential to stationary phase, suggesting that despite the delayed start, the growth dynamics in later phases were comparable between the strains (Fig. 1C).

Colony morphology was evaluated according to previously established criteria for mycobacterial surface architecture (Ojha et al., 2008; Recht and Kolter, 2001). Consistent with prior reports, colonies of *M. avium* WT and Ms_2054 exhibited wrinkled, textured surfaces characteristic of enhanced surface complexity. Ms_2054 displayed a more pronounced wrinkled morphology compared to Ms_Vc. Similarly, the Δ2054 formed smooth colonies lacking the characteristic structure observed in *M. avium* WT (Fig. 1E). These results suggest that MAV2054 expression contributes to altered surface morphology, potentially by enhancing extracellular matrix production or promoting cell–cell interactions.

Previous studies have shown that several bacterial proteins contribute to resistance against extracellular stress (Ruan et al., 2020; Xu et al., 2023; Yang et al., 2021). To determine whether MAV2054 is involved in such responses, we exposed Ms_2054 and Ms_Vc to 0.05% SDS and acidic pH (pH 3). Under these conditions, no significant differences in survival were observed between the two strains (Fig. 1F and 1G). In addition, we measured the MIC values of *M. avium* and *M. smegmatis* strains to HCl and SDS. Under these conditions, both Ms_Vc and Ms_2054 exhibited identical MIC values (HCl: 0.125%; SDS: 0.025%), which were also comparable to those of *M. avium* WT and the Δ2054. Furthermore, we assessed susceptibility to clarithromycin (CLA), which is commonly used in the treatment of mycobacterial infections in clinical field. The MIC of CLA was 1 µg/ml for *M. avium* strains and 2 µg/ml for *M. smegmatis*, with no difference between MAV2054-expressing and control strains (data not shown). These findings indicate that MAV2054 expression does not confer a survival advantage under extracellular stress conditions.

In summary, although MAV2054 does not appear to affect extracellular stress resistance in *M. avium* and *M. smegmatis*, its expression significantly alters colony morphology, inducing a wrinkled phenotype. These findings suggest that MAV2054 may modulate mycobacterial surface characteristics, potentially enhancing aggregation or interactions with the host environment.

### Recombinant MAV2054 protein binds to the surface of host epithelial cells

A previous study reported that recombinant MAV2054 protein localizes within macrophages (Lee et al., 2016). Additionally, several studies have demonstrated that bacterial membrane proteins play crucial roles in binding to host cell surfaces (Bottomley et al., 2020; Choi et al., 2008; Lo and Sorensen, 2007; Tsaplina and Bozhokina, 2021). Based on these findings, we hypothesized that MAV2054 may also interact with host epithelial cell surfaces.

**Fig. 2.**
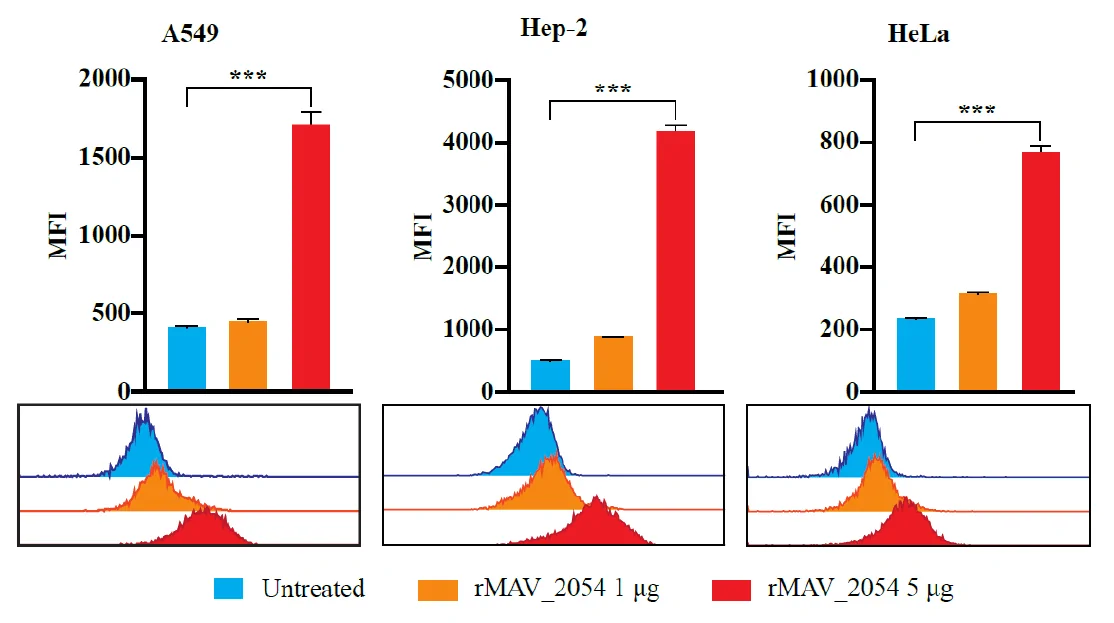
Dose-dependent binding of recombinant MAV2054 (rMAV_2054) to epithelial cell surfaces. Epithelial cells were incubated with increasing concentrations of rMAV_2054 (1 μg/ml and 5 μg/ml), followed by staining with a polyclonal anti-MAV2054 antibody and an Alexa Fluor 488-conjugated secondary antibody. Flow cytometry histograms show background fluorescence (blue-shaded region) from cells stained without rMAV2054 (negative control), and increased fluorescence signals from cells treated with 1 μg/ml (orange) and 5 μg/ml (red) rMAV2054. Data are presented as mean ± SEM. Statistical significance was determined using one-way ANOVA with ^***^p < 0.001. rMAV_2054, recombinant MAV2054 protein.

To test this hypothesis, the binding ability of recombinant MAV2054 (rMAV_2054) was evaluated using three human epithelial cell lines: A549, HEp-2, and HeLa. Flow cytometric analysis showed that rMAV_2054 binding to these cells increased in a dose-dependent manner (Fig. 2). These results suggest that MAV2054 is capable of directly interacting with the surface of host epithelial cells. Among the three tested cell lines, A549 cells were selected for further studies due to their relevance to pulmonary infection, which represents the primary clinical manifestation of NTM diseases, and their consistent susceptibility to bacterial invasion (To et al., 2020).

### MAV2054 promotes epithelial cell invasion and induces host membrane remodeling

**Fig. 3.**
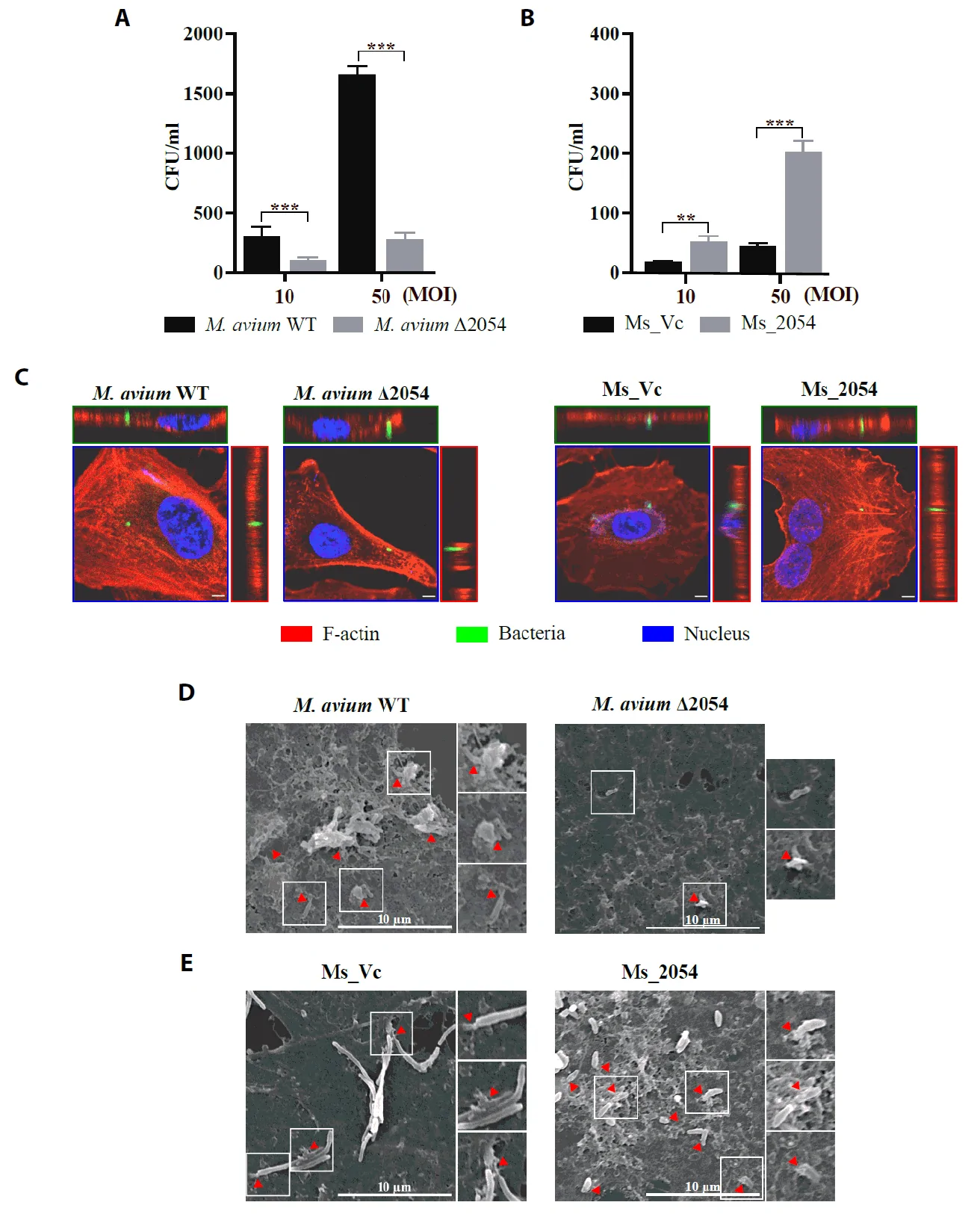
MAV2054 enhances mycobacterial adhesion and invasion of epithelial cells. (A) Invasion of *M. avium* Δ2054 into A549 epithelial cells. Cells were infected with *M. avium* WT or Δ2054 at MOIs of 10 and 50 for 6 h. Intracellular bacterial loads were quantified by serial dilution and plating. (B) Invasion of recombinant *M. smegmatis* strains (Ms_Vc and Ms_2054) into A549 epithelial cells at MOIs of 10 and 50 for 6 h. Intracellular bacterial numbers were quantified as described in (A). (C) Confocal fluorescence microscopy z-stack imaging of A549 cells infected with CFSE-labeled bacteria (green). Cells were infected with CFSE-labeled *M. avium* WT, Δ2054, Ms_2054, or Ms_Vc for 6 h at MOI of 10. Actin was stained with Alexa Fluor 594 Phalloidin (red), and nuclei with DAPI (blue). Orthogonal projections (x–z and y–z planes) are shown in red and green lines, respectively. Z-stack images were acquired at 7.0-µm sections. Scale bars, 1 μm. (D, E) Scanning electron microscopy images of A549 cells infected with *M. avium* (D) or *M. smegmatis* (E) strains. Red arrows indicate membrane rearrangements associated with bacterial invasion. Scale bars, 10 μm and 5 μm, respectively. Data are presented as mean ± SEM. Statistical significance: ^**^p < 0.01, ^***^p < 0.001. WT, *M. avium* wild-type; Δ2054, *M. avium* MAV_2054 deletion mutant; Ms_Vc, *M. smegmatis* vector control; Ms_2054, MAV_2054-expressing *M. smegmatis*.

To further explore the functional consequences of MAV2054–host interactions, we investigated whether MAV2054 expression enhances bacterial attachment and invasion. Invasion assays were performed by quantifying intracellular bacterial loads in A549 cells. At an MOI of 10 or 50, *M. avium* WT strains exhibited significantly higher intracellular bacterial burdens compared to the Δ2054 (Fig. 3A). Similarly, the Ms_2054 strain showed significantly higher intracellular bacterial loads than the vector control strain (Ms_Vc), indicating that ectopic expression of MAV2054 increases the invasive capacity of mycobacteria (Fig. 3B). These consistent findings across two distinct *Mycobacterium* species support a direct role for MAV2054 in promoting host cell entry.

To further characterize the interaction between bacteria and host cells, we conducted microscopic analyses. Confocal Z-stack imaging revealed that both Ms_2054 and *M. avium* WT strains were localized within phalloidin-stained actin structures, indicating successful internalization and association with the host cytoskeleton. In contrast, the Δ2054 and Ms_Vc strains were predominantly observed outside actin-rich regions, suggesting impaired or absent cellular entry (Fig. 3C).

Scanning electron microscopy confirmed bacterial attachment to the surface of A549 cells under all infection conditions. Notably, membrane protrusions and ruffling were prominently observed in cells infected with Ms_2054 or *M. avium* WT (marked by red arrows). In contrast, these features were infrequently observed in cells infected with the Δ2054 or Ms_Vc strains, indicating that MAV2054 expression is closely associated with the induction of host membrane remodeling during bacterial entry (Fig. 3D and 3E). Collectively, these results demonstrate that MAV2054 facilitates epithelial cell invasion by enhancing bacterial adhesion, promoting entry into the host cytoskeleton, and inducing membrane remodeling at the host–pathogen interface.

### MAV2054 promotes pulmonary infection and host response in vivo

**Fig. 4.**
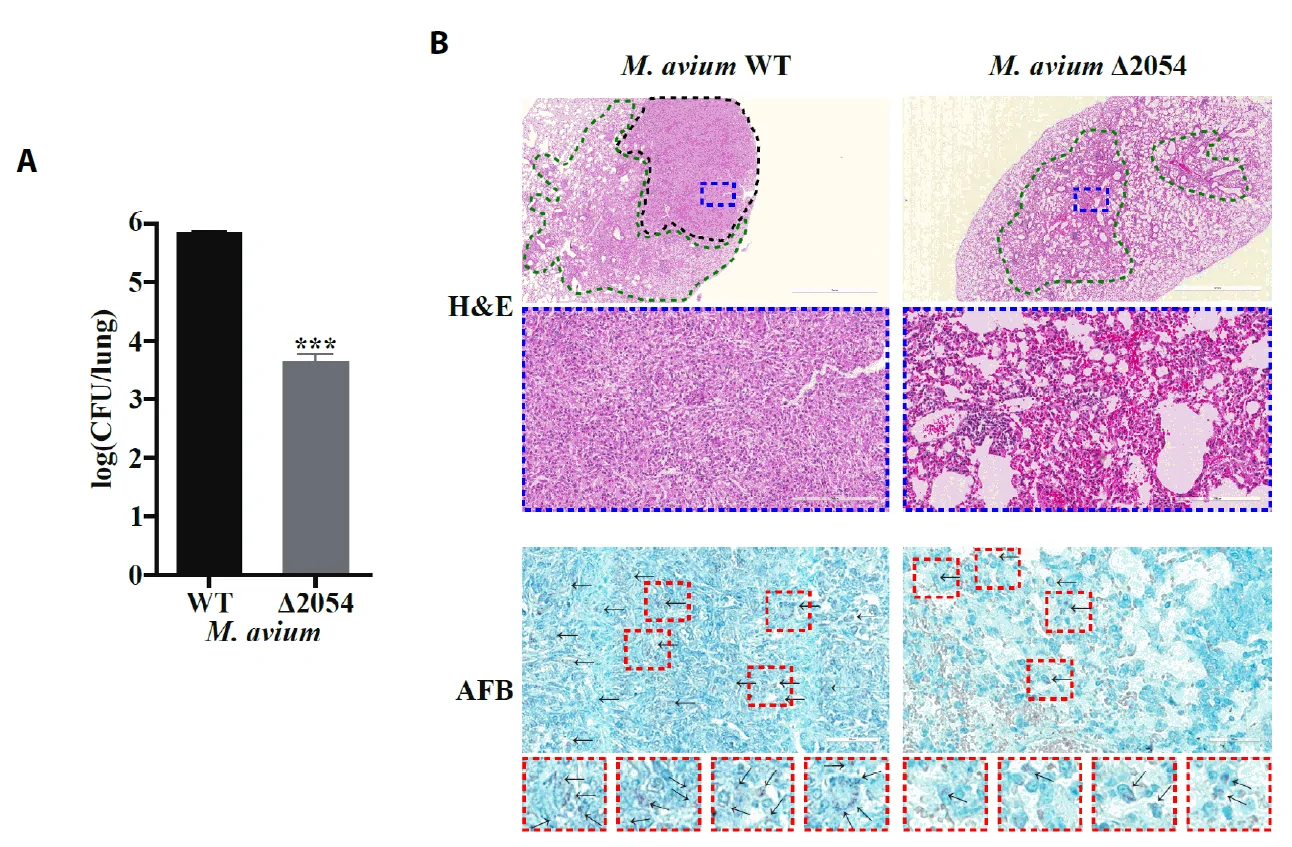
MAV2054 enhances *M. avium* survival in a murine pulmonary infection model. (A) Bacterial burden in the lungs of mice 2 weeks after intratracheal infection with *M. avium* WT or Δ2054 strains. (B) Representative histopathological images of lung tissue. AFB-stained sections show AFB-positive bacilli (black arrows). H&E-stained sections shows unstructured neutrophilic infiltration (black dotted lines area) and cellular inflammatory lesions (green dotted lines area). Data represent one of two independent experiments (n = 3 mice per group) and are presented as mean ± SEM. Statistical significance: ^***^p < 0.001. WT, wild-type; Δ2054, *M. avium* MAV2054 deletion mutant; Acid-fast bacilli, AFB; Hematoxylin and eosin, H&E.

To assess the role of MAV2054 in *M. avium* infection in vivo, mice were intratracheally infected with either the *M. avium* WT strain or the Δ2054. Two weeks post-infection, bacterial burden and lung pathology were evaluated. Mice infected with the *M. avium* Δ2054 exhibited significantly lower bacterial loads in the lungs compared to those infected with the WT strain (Fig. 4A). Furthermore, numerous AFB were seen in the lungs of mice infected with *M. avium* WT while few AFB-stained lung was observed from mice infected with Δ2054 (Fig. 4B).

Histological analysis using H&E staining revealed that *M. avium* WT infection induced marked pulmonary inflammation, characterized by the formation of unstructured neutrophilic infiltration (black dotted lines area; 1.059) and cellular inflammatory lesions (green dotted lines area; 1.635). In contrast, lungs from mice infected with Δ2054 exhibited mild inflammation, with only cellular inflammatory lesions (green dotted lines area; 0.811 and 0.153), and unstructured neutrophilic infiltration were not detected (Fig. 4B). Collectively, these results indicate that MAV2054 contributes to epithelial cell invasion and plays a critical role in the in vivo pathogenicity of *M. avium*.

### MAV2054 promotes Cdc42 activation to support host cell entry

**Fig. 5.**
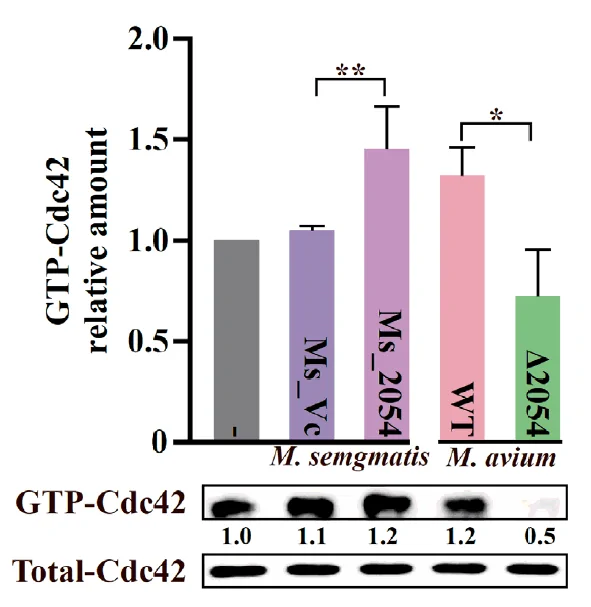
MAV2054 activates Cdc42 during mycobacterial infection. Quantification of Cdc42 activation in A549 cells following infection with *M. avium* or recombinant *M. smegmatis* strains at a MOI of 10 for 1 h. Cdc42 activity was measured using a pull-down assay. Data are presented as mean ± SEM. Statistical significance: ^*^p < 0.05, ^**^p < 0.01. WT, *M. avium* wild-type; Δ2054, *M. avium* MAV2054 deletion mutant; Ms_Vc, *M. smegmatis* vector control; Ms_2054, MAV2054-expressing *M. smegmatis*.

*Campylobacter jejuni* (Krause-Gruszczynska et al., 2007) and *Salmonella* (Bandyopadhyay et al., 2024) are known to promote host cell entry by activating Cdc42, a Rho family GTPase that regulates actin cytoskeletal dynamics (Farrugia and Calvo, 2017). To determine whether MAV2054-mediated bacterial invasion involves Cdc42 activation, we performed a Cdc42 pull-down assay. The results revealed that Ms_2054 induced higher levels of active Cdc42 compared to the vector control strain Ms_Vc. Similarly, *M. avium* WT strains exhibited greater Cdc42 activation than the Δ2054 (Fig. 5). These findings suggest that MAV2054 promotes bacterial invasion by activating Cdc42, a key regulator of actin cytoskeletal remodeling required for host cell entry.

### MAV2054-mediated invasion requires actin cytoskeletal remodeling

**Fig. 6.**
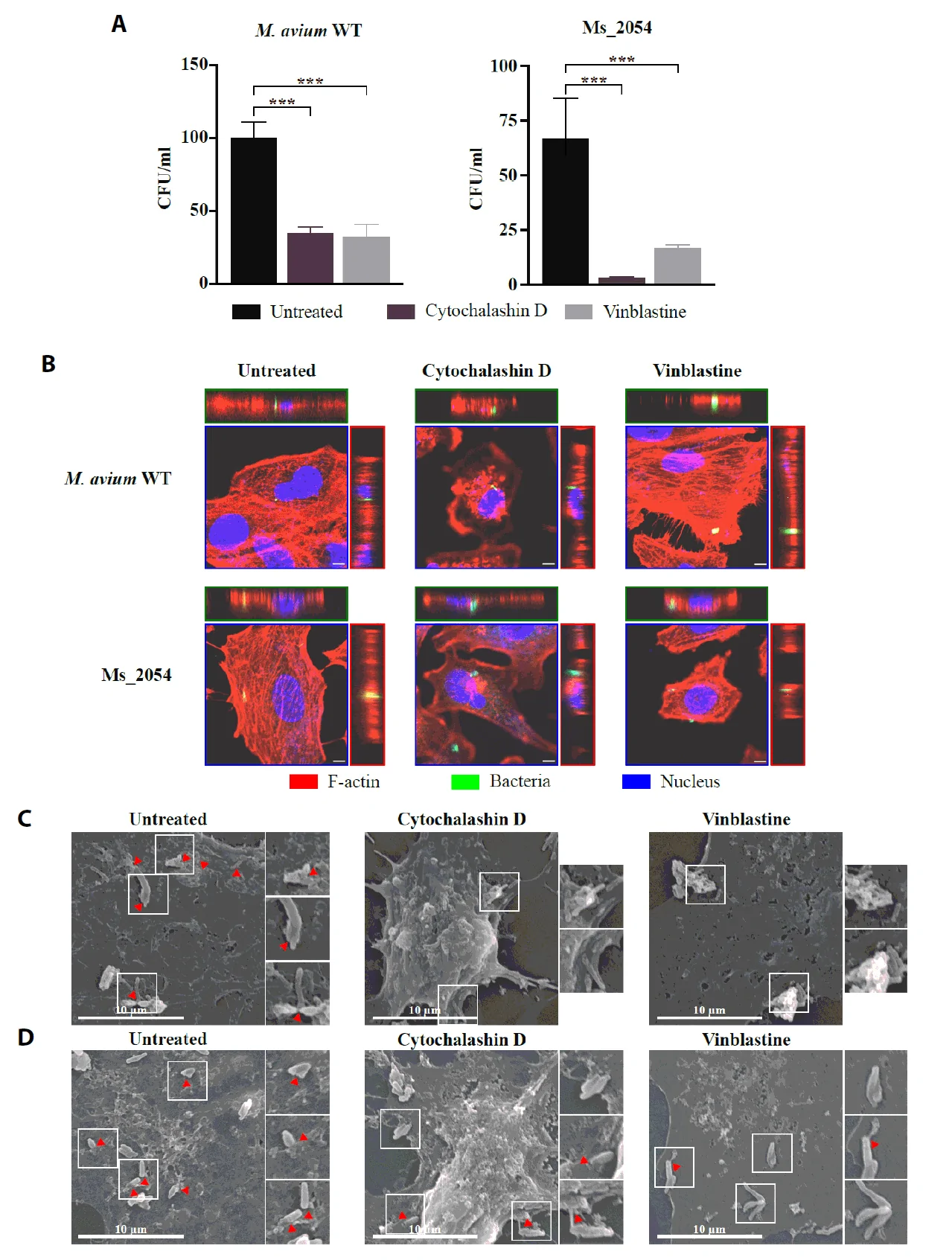
MAV2054-mediated bacterial invasion is inhibited by cytoskeletal disruption. A549 cells were pretreated with cytochalasin D (actin polymerization inhibitor) or vinblastine (microtubule polymerization inhibitor) for 30 min, followed by infection with *M. avium* WT or Ms_2054 at a MOI of 10 for 6 h. (A) Quantification of intracellular *M. avium* WT (left) and Ms_2054 (right) in the presence of cytoskeletal inhibitors. (B) Confocal z-stack fluorescence imaging of infected A549 cells. Cells were pretreated with inhibitors and infected with CFSE-labeled (green) bacteria. Z-stack images were captured at 7.0-µm sections; red and green lines indicate orthogonal views (y–z and x–z planes). F-actin was stained with Alexa Fluor 594 phalloidin (red) and nuclei with DAPI (blue). Scale bars, 1 μm. (C, D) Scanning electron microscopy images of A549 cells infected with *M. avium* WT (C) or Ms_2054 (D) in the presence of cytoskeletal inhibitors. Red arrows indicate membrane rearrangements associated with bacterial entry. Scale bars, 10 μm and 5 μm, respectively. Data are presented as mean ± SEM. Statistical significance: ^***^p < 0.001. WT, *M. avium* wild-type; Ms_2054, MAV2054-expressing *M. smegmatis*.

Given the role of Cdc42 in regulating actin polymerization, we next examined whether MAV2054-mediated bacterial invasion depends on cytoskeletal remodeling. Actin polymerization is a well-established prerequisite for the formation of membrane protrusions, such as ruffles and filopodia-like structures, which facilitate bacterial internalization (Lee et al., 2010; Mahankali et al., 2011). To test this, epithelial cells were pretreated with cytoskeletal inhibitors prior to infection. Specifically, cytochalasin D (which disrupts microfilaments) and vinblastine (which targets microtubules) were used. Both treatments significantly reduced the intracellular bacterial load of the Ms_2054 strain, indicating that cytoskeletal dynamics are essential for invasion. Similarly, a marked reduction in intracellular bacterial burden was observed for the *M. avium* WT strain in the presence of either inhibitor (Fig. 6A). Consistent with these findings, confocal Z-stack imaging revealed that Ms_2054 and *M. avium* WT strains were predominantly localized outside actin-rich regions when cells were pretreated with either inhibitor, indicating impaired internalization due to disrupted cytoskeletal architecture (Fig. 6B). Furthermore, scanning electron microscopy analysis supported this observation: membrane rearrangements, such as ruffling and protrusions, which were prominent in cells infected with *M. avium* WT or Ms_2054, were infrequently detected in inhibitor-treated cells (Fig. 6C and 6D). These results suggest that MAV2054 facilitates bacterial entry into epithelial cells via an actin polymerization-dependent mechanism. 

## Discussion

In this study, we demonstrated that MAV2054, a protein from *M. avium*, plays a pivotal role in promoting bacterial invasion of host epithelial cells. Through genetic manipulation via overexpression and deletion, we showed that disruption of MAV2054 significantly reduced *M. avium* invasion into A549 cells, while heterologous expression in *M. smegmatis* significantly enhanced invasion efficiency. These findings strongly support the role of MAV2054 as a virulence-associated factor critical for host–pathogen interaction.

Interestingly, a growth difference was observed between *M. avium* WT and the Δ2054, suggesting that MAV2054 may contribute to strain-specific growth, potentially through effects on stress response or metabolic regulation unique to *M. avium*. In contrast, no significant growth difference was seen between Ms_2054 and Ms_Vc, indicating that MAV2054 does not influence general in vitro growth in *M. smegmatis*. This discrepancy likely reflects intrinsic genetic and physiological differences between the two mycobacterial species. Importantly, these findings confirm that the enhanced invasion seen in Ms_2054 is not due to increased bacterial proliferation but rather due to MAV2054-specific virulence activity. A similar decoupling of growth and virulence has been described for the ESX-1 secretion system in *M. tuberculosis*, which is essential for virulence but dispensable for in vitro growth (Simeone et al., 2009).

Mechanistically, we found that MAV2054 expression was associated with increased activation of Cdc42, a Rho family GTPase critical for actin cytoskeletal remodeling (Watson et al., 2017). This suggests that MAV2054 may trigger intracellular signaling pathways involved in membrane remodeling and bacterial uptake. Treatment with cytoskeletal inhibitors such as cytochalasin D and vinblastine impaired invasion, indicating that MAV2054-mediated entry is dependent on host cytoskeletal remodeling.

While both cytochalasin D and vinblastine significantly reduced MAV2054-mediated invasion, their mechanisms of action differ—cytochalasin D inhibits actin polymerization, whereas vinblastine disrupts microtubule assembly (Menanteau-Ledouble et al., 2018). Despite targeting distinct cytoskeletal elements, both inhibitors impaired bacterial entry to a similar extent, suggesting that MAV2054-driven invasion depends on coordinated cytoskeletal remodeling. Interestingly, previous studies have demonstrated that vinblastine inhibits bacterial internalization in systems such as *Enterobacter sakazakii* and *Yersinia ruckeri*, likely through indirect modulation of host cytoskeletal dynamics (Menanteau-Ledouble et al., 2018). In *Campylobacter jejuni*, vinblastine did not block autophagy-associated invasion enhancement, whereas cytochalasin D completely abolished it, underscoring the central role of actin (Fukushima et al., 2022). Taken together, these findings suggest that the inhibitory effect of vinblastine observed in our study may reflect a secondary or indirect mechanism—possibly through disruption of actin–microtubule crosstalk or trafficking of key host factors. While actin polymerization appears to be the principal driver of MAV2054-mediated invasion, microtubule integrity may still be required for efficient host cytoskeletal coordination. Further studies are warranted to clarify these interactions.

Although MAV2054 expression correlated with Cdc42 activation, the precise molecular mechanism underlying this pathway remains unclear. Similar strategies have been observed in other bacterial pathogens; for example, IpgB1 of *Shigella* promotes membrane ruffling via activation of Cdc42 and Rac1 (Ohya et al., 2005), and the *Escherichia coli* type III effector Map functions as a guanine nucleotide exchange factor for Cdc42 (Huang et al., 2009). These observations raise the possibility that MAV2054 may indirectly modulate upstream GTPase regulators or interact with host signaling complexes. Further studies are warranted to elucidate these mechanisms.

Our data also showed that while all bacterial strains adhered to the host cell surface, only Ms_2054 and *M. avium* WT were efficiently internalized, as confirmed by confocal microscopy and quantification of intracellular bacterial loads. This highlights that surface binding alone is insufficient for invasion, and underscores the importance of MAV2054-mediated cytoskeletal remodeling.

Notably, MAV2054 has recently been annotated as an encapsulin-like protein. Encapsulins are protein-based nanocompartments found in bacteria and archaea, typically involved in iron homeostasis and oxidative stress response (Giessen, 2016). Emerging evidence suggests that certain encapsulins may also modulate host–pathogen interactions (Andreas and Giessen, 2021). Our findings extend this concept by demonstrating that MAV2054, a family 2A encapsulin nanocompartment shell protein, directly contributes to epithelial cell invasion and host signaling modulation. This uncovers a previously unrecognized role for encapsulin-like proteins in bacterial virulence beyond their conventional metabolic functions.

While most previous studies have focused on the intracellular survival of *M. avium* within macrophages (Early et al., 2011), our study provides novel insights into the molecular mechanisms of epithelial cell invasion, an early but underexplored event in pulmonary pathogenesis. However, this study has some limitations. The specific host receptors and signaling intermediates interacting with MAV2054 remain unidentified. In addition, our experiments were limited to epithelial cell lines, and it remains unclear whether MAV2054 exerts similar effects in macrophages or in vivo. Future studies investigating host targets and structural features of MAV2054 will be critical to fully understand its role in both invasion and bacterial adaptation.

In conclusion, we propose that MAV2054 functions as both a structural component of a metabolic nanocompartment and a virulence factor, promoting epithelial cell invasion through cytoskeletal remodeling. These findings expand our understanding of encapsulin functions and offer novel therapeutic targets for mitigating *M. avium* pathogenesis. To our knowledge, this is the first study to implicate Cdc42 activation in the context of *M. avium* infection, highlighting a previously unrecognized mechanism of host cell invasion by MAC organisms.
